# Development and Application of High-Content Biological Screening for Modulators of NET Production

**DOI:** 10.3389/fimmu.2018.00337

**Published:** 2018-03-05

**Authors:** Ilaria J. Chicca, Michael R. Milward, Iain Leslie C. Chapple, Gareth Griffiths, Rod Benson, Thomas Dietrich, Paul R. Cooper

**Affiliations:** ^1^School of Dentistry, Institute of Clinical Sciences, University of Birmingham, Birmingham, United Kingdom; ^2^Imagen Therapeutics Ltd., Manchester, United Kingdom

**Keywords:** neutrophil extracellular traps, high-content analysis, high-content screening, nuclear decondensation, MAPK, Src, mTOR, glutathione reductase

## Abstract

Neutrophil extracellular traps (NETs) are DNA-based antimicrobial web-like structures whose release is predominantly mediated by reactive oxygen species (ROS); their purpose is to combat infections. However, unbalanced NET production and clearance is involved in tissue injury, circulation of auto-antibodies and development of several chronic diseases. Currently, there is lack of agreement regarding the high-throughput methods available for NET investigation. This study, therefore, aimed to develop and optimize a high-content analysis (HCA) approach, which can be applied for the assay of NET production and for the screening of compounds involved in the modulation of NET release. A suitable paraformaldehyde fixation protocol was established to enable HCA of neutrophils and NETs. Bespoke and in-built bioinformatics algorithms were validated by comparison with standard low-throughput approaches for application in HCA of NETs. Subsequently, the optimized protocol was applied to high-content screening (HCS) of a pharmaceutically derived compound library to identify modulators of NETosis. Of 56 compounds assessed, 8 were identified from HCS for further characterization of their effects on NET formation as being either inducers, inhibitors or biphasic modulators. The effects of these compounds on naïve neutrophils were evaluated by using specific assays for the induction of ROS and NET production, while their modulatory activity was validated in phorbol 12-myristate 13-acetate-stimulated neutrophils. Results indicated the involvement of glutathione reductase, Src family kinases, molecular-target-of-Rapamycin, and mitogen-activated-protein-kinase pathways in NET release. The compounds and pathways identified may provide targets for novel therapeutic approaches for treating NET-associated pathologies.

## Introduction

Neutrophils are key components of the innate immune response and migrate to infected peripheral tissues to combat invading microorganisms (1). Well-known antimicrobial strategies employed by neutrophils include phagocytosis, degranulation of antimicrobial peptides and reactive oxygen species (ROS) (2). However, another strategy neutrophils utilize for clearing or limiting infections in tissues is *via* their release of neutrophil extracellular traps (NETs) (3).

The formation and physiological function of NETs were initially described as a neutrophil antimicrobial strategy targeted against both Gram-positive and Gram-negative bacteria (3). NETs comprise extracellular deoxyribonucleic acid (DNA)-fibers that form web-like structures that immobilize pathogens, arresting their spread within tissues and, eventually, facilitating their death. Fundamental steps in NET formation include the production of ROS, the de-condensation of nuclear chromatin modulated by the peptidylarginine deiminase 4 enzyme, the mixing of DNA with granule proteins and, finally, the active extrusion of the NETs through the outer cell membrane (4, 5).

Neutrophil extracellular trap structures comprise a DNA backbone decorated with neutrophil granule proteins most of which have been reported to have antimicrobial properties, including histones, neutrophil elastase, and myeloperoxidase (MPO) (3, 6). Notably, ineffective NET formation *in vivo* can result in pathogen survival and infection spread, as reported in chronic granulomatous disease (CGD) patients (4). Beside the beneficial antimicrobial function of NETs; however, the presence of DNA/protein complexes in extracellular tissue has been associated with several pathologies. Excessive production and/or delayed or defective removal of NETs are understood to play a role in the pathogenesis of inflammatory diseases, such as cystic fibrosis (CF) and systemic lupus erythematosus (SLE) (7–9). Furthermore, NET structures have been implicated in thrombus formation in deep vein thrombosis (DVT) (10), in cancer development (11) and rheumatoid arthritis (RA) (12, 13). In recent years, an increasing number of inflammatory conditions have been proposed to be associated with NET production including periodontitis, which is a chronic inflammatory disease triggered by an imbalanced bacterial colonization and host response (14). Due to their contradictory functions in human biology and disease, NETs have been described as being a “double-edged sword” and their homeostatic control may determine the difference between health and disease (15).

Neutrophil extracellular trap assays are frequently based on the identification of their components, including DNA or NET-related proteins, with levels determined by immunostaining, immunoblotting, or ELISA (5, 16–18). These methods can be low-throughput, and, consequently, are time consuming and/or expensive. Furthermore, the application of different techniques may contribute to the lack of consistency in findings in the field and fuel the controversies raised by several authors regarding the validity of some of the previously reported results (19, 20). Subsequently, in recent years several alternative approaches have been developed to enable relatively high-throughput analysis of NETs. Brinkmann et al. (21) published a semi-automated method for image-based quantification of NETs using nucleus area and chromatin staining intensity. Moussavi-Harami et al. (22) also developed a microfluidic device for the quantification of ROS and NETs which utilized whole blood samples and Gavillet et al. (23) reported a flow-cytometry based quantification approach. Interestingly, Zhao et al. (24) proposed a new technique for the identification of suicidal and vital NET release based on the integrated use of flow-cytometry and image analysis.

In this paper, we report the development of an innovative solution for analyzing NETs using high-content analysis (HCA), which combines automated high-throughput imaging with automated computational data analysis (25). The technology has been extensively employed in industry for compound lead identification and drug discovery processes. Although the technology has plenty of application in cell biology (26, 27), this study aims for the first time to validate HCA for automatic characterization of NETosis. The main advantages of HCA include its ability to quantify protein staining intensity, determination of molecular subcellular localization, independent cell information, and the rapid acquisition of multiparametric data, such as information on spatial and temporal variables (28). The investigation of biological features is performed using high-resolution image capture by an automated fluorescence microscope capable of reading 96-, 384-, 1536-well plate formats, which dramatically increases the number of conditions that can be analyzed simultaneously (29). A notable advantage of HCA compared with previously reported high-throughput technologies for NETs is the capture of data from the entire well. Using a completely automated image-capture and image-interpretation system, the data obtained by HCA represents the average biological response calculated from the entire population of cells interacting with the stimulus. This feature will notably eliminate user bias from the interpretation of neutrophil responses and can potentially address several of the controversies raised regarding NET studies (20, 21).

Studies are now focusing on the identification of compounds able to pharmacologically modulate NET production for use in the treatment of NET-related diseases (30–33). Cell-based HCA has been extensively employed for the screening of compound libraries, often referred to as high-content screening (HCS), which aims to identify novel therapeutic leads (34). Here, we report a completely automated method for the analysis and quantification of NETs based on monitoring nuclear de-condensation and extracellular DNA release using HCA and apply this for HCS of a pharmaceutically derived compound library.

## Materials and Methods

### Neutrophil Preparation

Venous blood was collected from healthy volunteers (Ethical approval number: 10/H1208/48) and peripheral blood neutrophils were isolated using Percoll density gradients and subsequent red blood lysis as previously described (35). Neutrophil stimulation was induced by exposing cells to 50 nM Phorbol 12-myristate, 13-acetate (PMA; Sigma P8139) for up to 4 h and inhibited by treating cells for 30 min, prior to PMA stimulation, with 10 µM Celastrol (Cayman 70950), which is a well-characterized NET inhibitor (33).

### Paraformaldehyde Fixation of NETs

A 20% paraformaldehyde solution (Sigma P6148) was added directly to the neutrophil culture medium providing a final concentration of 4% paraformaldehyde. Subsequently, samples were incubated for 10 min at room temperature. Plates were then centrifuged for 10 min at 1,800 rcf, medium was carefully removed and the resulting fixed samples were washed twice with PBS.

### Compound Library Preparation

The 56 compounds were diluted in DMSO as per manufacturers’ instructions (detailed in Table S1 in Supplementary Material). Master plates with compounds at 1,000 times concentration were prepared in advance, sealed and stored at −20°C until use. Compounds were combined and diluted in PBS to obtain five concentrations ranging between 0.025 nM and 0.25 mM. 20 µl of each concentration were added to 30 µl of cell media containing 5 × 10^3^ neutrophils to obtain compound concentrations ranging between 0.01 nM and 100 µM.

### ROS Quantification

Neutrophils were diluted in glucose-supplemented PBS and seeded at 1 × 10^5^ cells/well in white 96-well plates (Costar 3912), pre-treated overnight with 1% bovine serum albumin (BSA; Sigma A4503) in PBS. ROS release was quantified by using an enhanced chemiluminescence assay as previously described in Matthews et al. (35). Neutrophils were incubated with 450 nM Luminol (Sigma A8511) in a luminometer (Berthold Tristar^2^ LB942) for 30 min before stimulation and further incubation for up to 3 h. Readings were obtained at 37°C as relative light units.

### Fluorometric Quantification of NET-DNA

Neutrophils were diluted in RPMI (Sigma R7509) and seeded at 1 × 10^5^ cells/well, unless otherwise specified, in clear 96-well plates (Costar 3370), pre-treated overnight with PBS 1% BSA. Fluorometric quantification of NET-DNA was performed according to the protocol previously described by Palmer et al. (5). Neutrophils were pre-incubated at 37°C in 5% CO_2_ for 30 min prior to stimulation and further incubation at 37°C in 5% CO_2_ for up to 4 h. Post-incubation samples were treated for 10 min with 1 unit/ml micrococcal nuclease (MNase; Worthington 4789), centrifuged for 10 min at 1,800 rcf and 150 µl of supernatant was transferred to a black 96-well plate (Costar 3915). NET-DNA was stained with 1 µM Sytox green^®^ (Invitrogen S7020) and quantified with a fluorometer (Berthold Twinkle^2^ LB970; ex:485 nm/em:525 nm). Readings were obtained at 37°C as arbitrary fluorescence units.

### HCA of NETs

Neutrophils were diluted in RPMI and seeded at 1 × 10^5^ cells/well, unless otherwise specified, in black, clear-bottom 96-well plates (Greiner Bio-one 655096), and pre-treated overnight with PBS 1% BSA. Neutrophils were pre-incubated at 37°C in 5% CO_2_ for 30 min prior to stimulation and further incubated at 37°C in 5% CO_2_ for up to 4 h. Post-incubation, neutrophils were fixed with 4% PFA and stained with 1 µM Sytox green^®^ or 20 µg/ml Hoechst 33342 (Sigma 14533).

### HCS of NETosis Modulators

Neutrophils were diluted in RPMI and 5 × 10^3^ cells were seeded in black, clear-bottom 96-well plates (Greiner Bio-one 781096). The 56-compound library, at the range of concentrations specified in Table S1 in Supplementary Material, was applied to the seeded neutrophils followed by 30 min incubation at 37°C in 5% CO_2_. Subsequently, where specified, NETs were induced with PMA as described above and following 3 h incubation, cells were fixed with 4% Formaldehyde and stained with 20 µg/ml Hoechst 33342.

### Image Acquisition for HCA and HCS

Samples were loaded onto the Cellomics™ ArrayScan™ (Thermo-Fisher Scientific VTI) and high-resolution images of 30 fields/well were acquired and captured using an automated excitation filter wheel and automated Observer Z1 inverted microscope. Operations were driven by the HCS Studio 2.0 Client Software (ThermoFisher Scientific VTI) using a range of feature settings for autofocus, auto exposure, and cell sub-population.

### Automated Image Analysis

Automated image analysis was performed using the ArrayScan HCS Reader software (Cellomics ThermoFisher) employing algorithms for Compartmental, Tube formation, NET detection or Nuclear decondensation analysis. Parameters for cell identification and exclusions were optimized for NETs or nuclei analysis (Imagen Therapeutics Ltd., UK).

### Statistics

Statistical analysis was performed using Prism versions 6 and 7 (GraphPad). Each experiment was performed in triplicate and on at least three occasions using cells from different healthy volunteers. Statistical tests were two-sided, with significance defined at α = 0.05.

## Results

### Identification of an NET Fixation Protocol Suitable for Use with HCA

**Figure 1:**
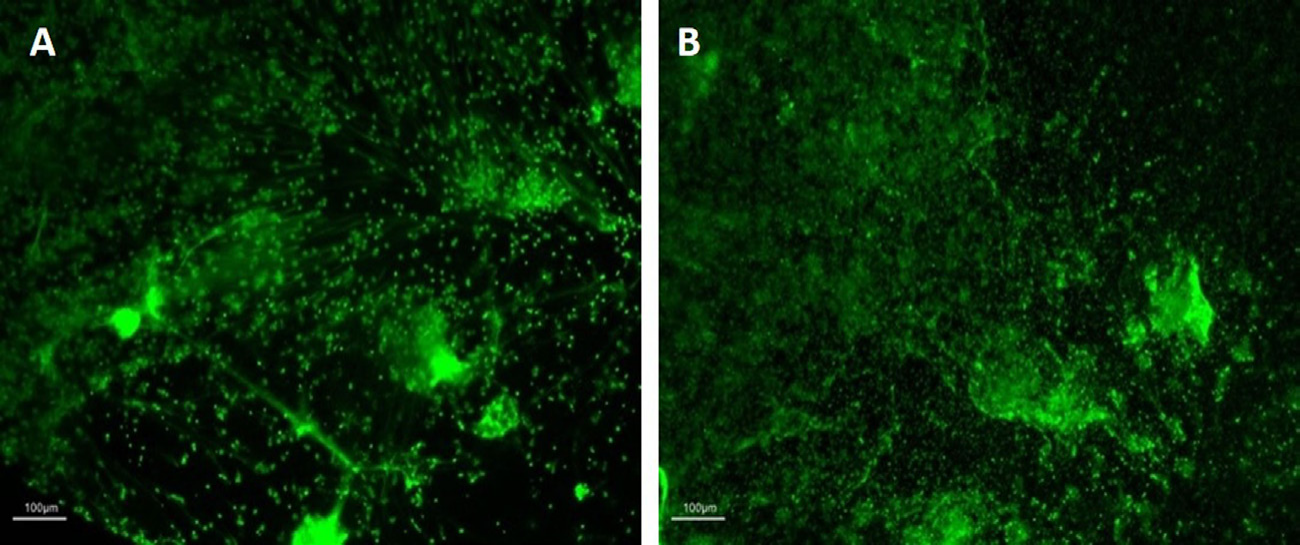
Images of neutrophil extracellular traps (NETs) before **(A)** and after **(B)** fixation with PFA. Images representative of independent experiments acquired before **(A)** and after **(B)** the fixation procedures. NETs were visualized at 10× magnification with fluorescence microscopy (Nikon Eclipse TE300) upon Sytox green staining. Scale bars are shown.

Investigating biological samples using HCA requires cell imaging for accurate and rapid examination which, in turn, requires fixation and appropriate staining to preserve cellular features (26). Previously reported neutrophil fixation protocols involve numerous wash steps prior to image acquisition and analysis (36). Notably, the mechanical action applied during these wash steps can result in the loss of sample material and, in particular, the disruption of NET structures. Subsequently, we have established an appropriate protocol for NET fixation using PFA in microplate formats in order to prevent material loss and artifact generation. Images of NETs before and after modifications of the fixation protocol were observed to identify their effect on sample integrity. Assay variations included applying in different orders of the fixation protocol the addition of PFA, wash steps, and centrifugation procedures. Following a qualitative assessment of the outcomes of the various fixation protocols (data not shown), we identified the most appropriate protocol as consisting of careful addition of PFA directly into stimulated NET cultures followed by centrifugation and washing with PBS as described in Section “Materials and Methods.” Representative images of the outcome pre- and post-fixation are provided in Figure 1. Notably the fixation agent directly interacts with the stimulated NETs potentially protecting them from further downstream mechanical disruption that may result from subsequent steps in the procedure. Differences between pre- and post-fixation are not easily identifiable indicating the fixation protocol successfully preserves sample integrity.

### Optimization and Validation of HCA Parameters for NET Analysis

**Figure 2:**
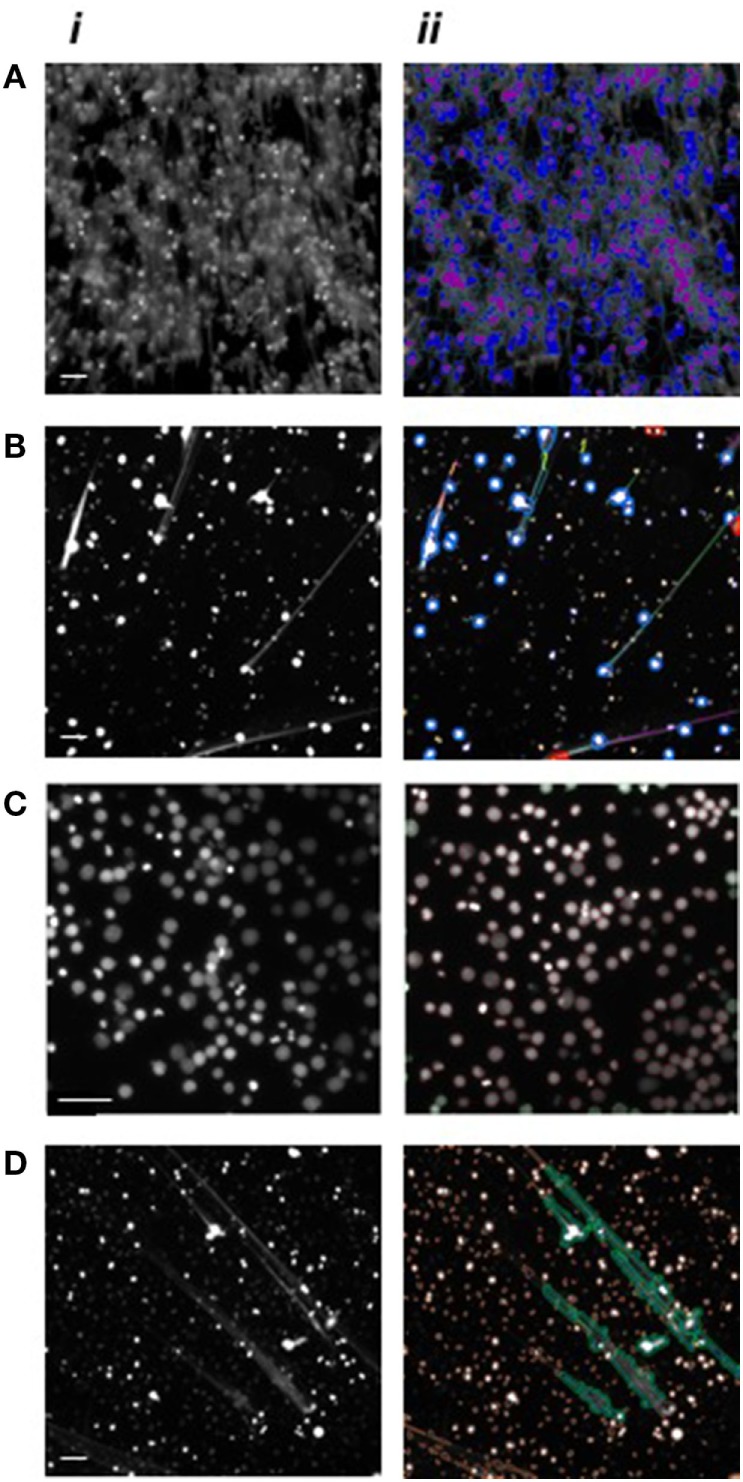
High-content analysis algorithm recognition of neutrophils and neutrophil extracellular traps (NETs). Images of PMA stimulated neutrophils following DNA fluorescence staining. Nuclei are shown in a decondensed state following stimulation and Hoechst staining while extracellular DNA is visible following Sytox green staining. (i) Images acquired using the ArrayScan, and (ii) applied automatic algorithm recognition. **(A)** Application of the Compartmental Analysis algorithm for the identification of neutrophil nuclei (purple) and NETs (blue). **(B)** Application of the Tube Formation algorithm for the identification of neutrophil nuclei (blue circles) and NETs (green, orange, yellow, and purple). **(C)** Application of the Nuclear Decondensation algorithm for the identification of neutrophil nuclei (circled in red). **(D)** Application of the NET Detection algorithm for the identification of NETs (green areas) while excluding intact cells (circled in orange). Scale bars represent 200 µm.

High-content analysis parameters were explored and optimized in order to develop an innovative high-throughput automated approach for NET analysis that utilized quantitative imaging for monitoring neutrophils, their nuclei and NET-related responses. Image analysis was performed using a bioinformatics software with built-in algorithms suitable for a diverse range of biological applications. Initially, two pre-existing algorithms, namely “Compartmental Analysis” and “Tube Formation,” were assessed for neutrophil and NET identification and analysis. NETs were induced by PMA stimulation and fixed with PFA. Neutrophil nuclei and NET structures were stained with Sytox green^®^. Images were acquired using the ArrayScan™ and analyzed using the HCS Reader software. NETs could potentially be recognized using the Compartmental Analysis algorithm, which quantifies the DNA in the circular space surrounding the nucleus (Figure 2A), or by the Tube Formation algorithm which quantifies DNA filaments extruded into the extracellular space (Figure 2B). Although NET quantification was effectively performed, we identified several limitations with these approaches including the lack of detection of early time-points of NET formation (data not shown). Therefore, two bespoke algorithms were developed for NET analysis and were termed “Nuclear Decondensation” and “NET Detection.” These algorithms were developed for the simultaneous monitoring of two fundamental characteristics of NET release, respectively, including nuclear decondensation (Figure 2C) and the subsequent release of DNA into the extracellular space (Figure 2D).

**Figure 3:**
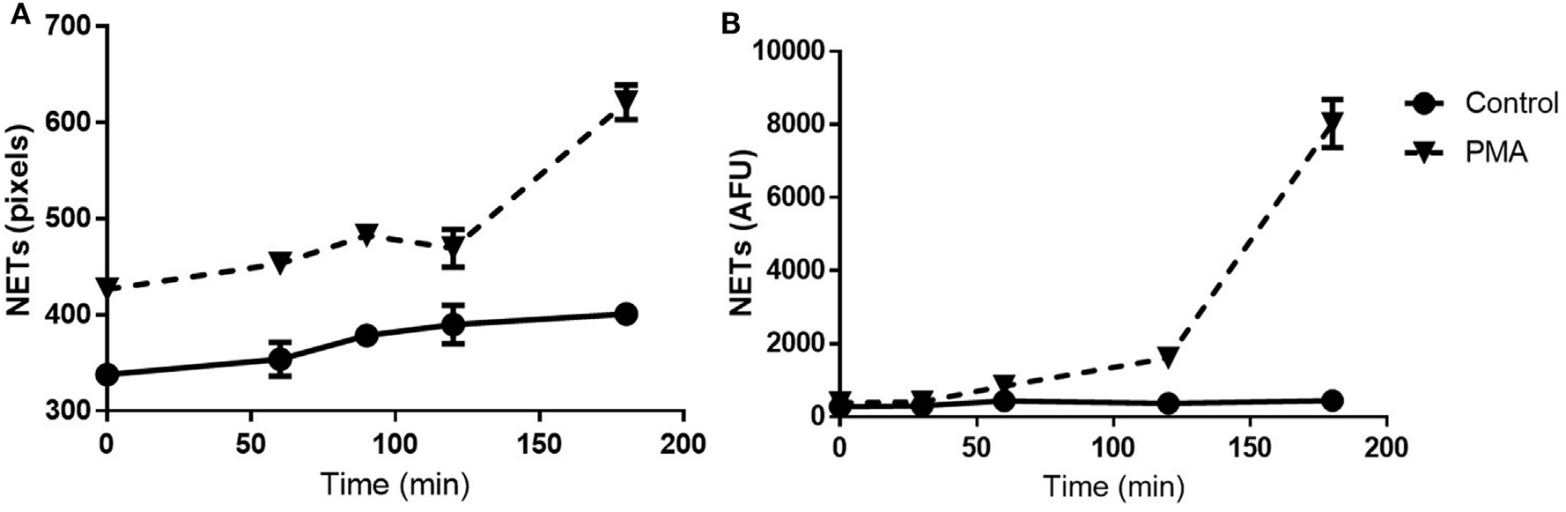
Time-course for neutrophil extracellular trap (NET) quantification: high-content analysis (HCA) vs fluorometric assay. Time-course of NET release in unstimulated and PMA-stimulated neutrophils up to 3 h. Comparison between HCA **(A)** and fluorometric **(B)** quantification. Values are expressed as units of fluorescent signal or arbitrary fluorescence units (AFU). Data represent duplicate means ± SEM.

The NET Detection algorithm was designed to identify filaments of extracellular DNA and to provide information on area, size, and intensity of those selected sections. Subsequently, we applied the NET Detection algorithm for the quantification of PMA-induced NET release over a 3-h time-course. Results indicated the technique detected NET units as early as 60 min after exposure to the stimulus, this detection then increased and peaked at 3 h. Interestingly, a relatively low amount of NETs were also identified immediately after PMA treatment. These data suggest HCA might be able to identify very early stages of NET formation as nuclear changes have been observed as rapidly as a few seconds after stimulation [data not shown and in Ref. (3, 4, 24)]. The algorithm also identified relatively low amounts of NETs in unstimulated neutrophils and this marginally increased by 90 min (Figure 3A). The accuracy of the HCA approach and data were further validated by comparison with a standard quantification method. NET analysis was performed by HCA and compared with standard fluorometric quantification, which determined levels of extracellular NET-DNA post-MNase digestion. Data obtained using the fluorometric quantification identified a similar trend in PMA-stimulated NET production and confirmed the validity of HCA data (Figure 3B).

**Figure 4:**
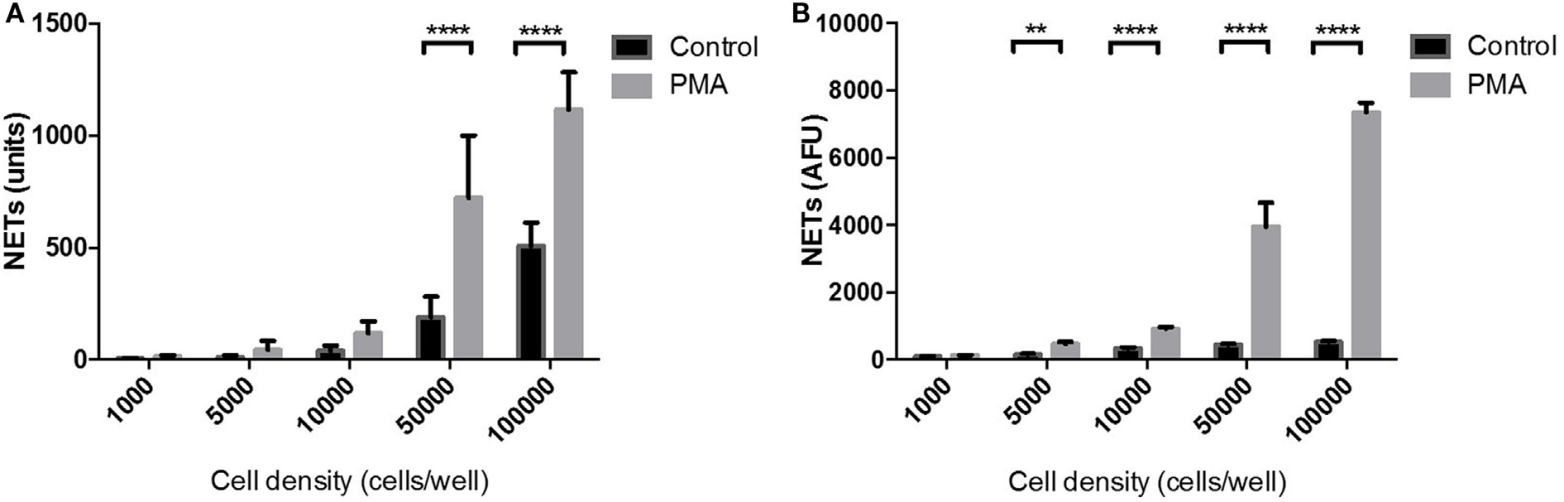
Neutrophil extracellular traps (NET) quantification in a range of cell densities: high-content analysis (HCA) vs fluorometric assay. Neutrophils seeded at a range of concentrations in a 96-well plate and stimulated for 3 h with PMA (gray bars) or unstimulated (black bars). NET quantification by HCA **(A)** and fluorometric assay **(B)**. Values are expressed as units of fluorescent signal or arbitrary fluorescence units (AFU) and as mean ± SEM. Statistical significance calculated using Sidak’s multiple comparison test (*n* = 3; **p < 0.01, ****p < 0.0001).

The sensitivity of the NET Detection algorithm was further evaluated using a range of neutrophil densities of between 1,000 and 100,000 cells/well which were subsequently stimulated or unstimulated with PMA for 3 h. The NET Detection algorithm outputs were compared, again, with data obtained by the standard fluorometric assay for validation purposes. The NET Detection algorithm registered significantly higher levels of NETs in PMA-stimulated compared with unstimulated neutrophils seeded at 50,000 cells/well and higher (Figure 4A). A similar trend of NET production was confirmed following fluorometric quantification, which also showed significant increases in PMA-induced NETs compared with controls in neutrophils seeded at 50,000 cells/well and above. This increase was also detected with as few as 5,000 neutrophils/well (Figure 4B). These validated results indicate that the HCA algorithm allowed sensitive and accurate NET quantification in samples with seeding densities above 50,000 neutrophils/well in a microplate format.

**Figure 5:**
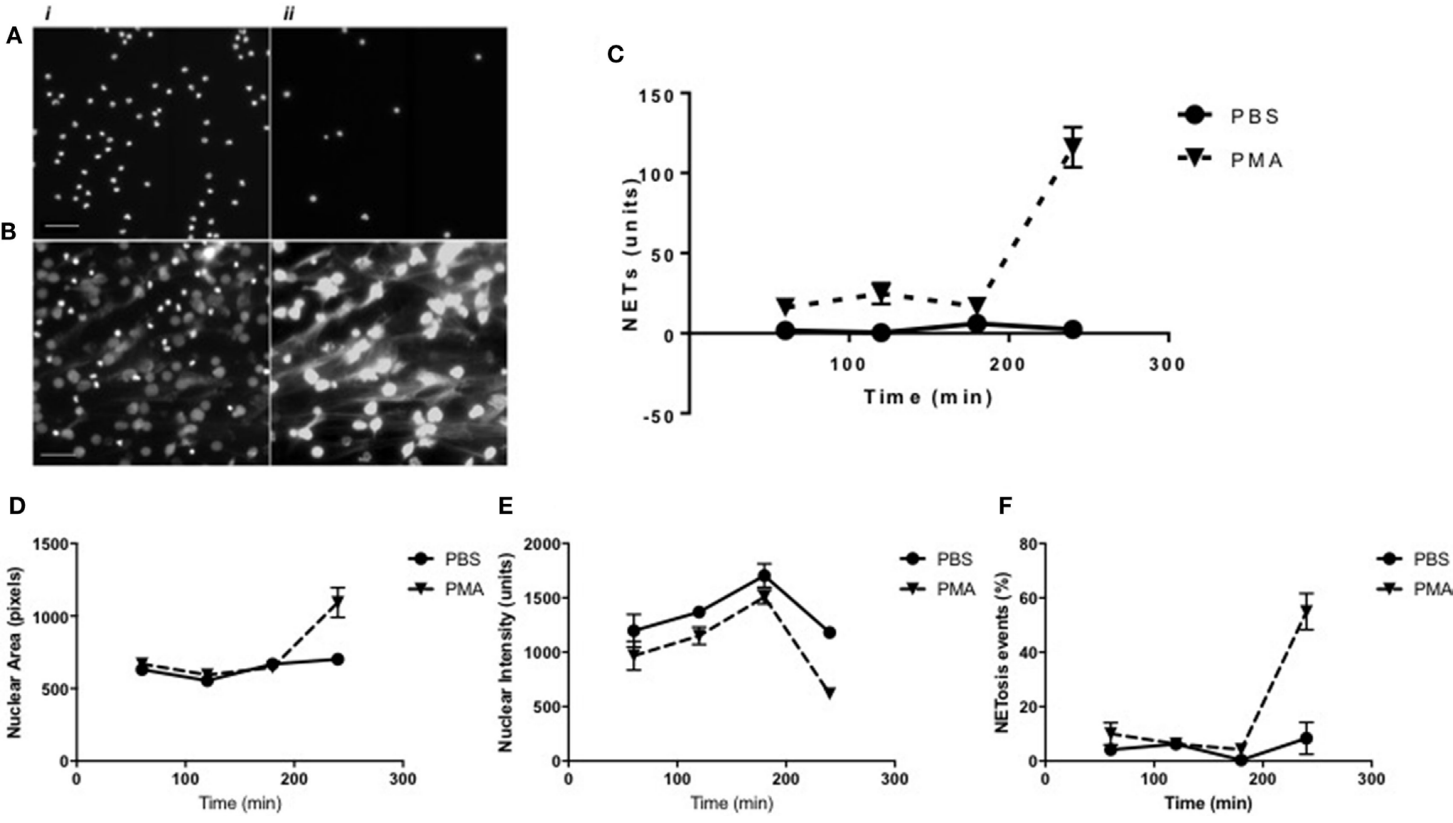
Time-course analysis of neutrophil extracellular trap (NET) formation as determined using the Nuclear Decondensation algorithm. Representative high-resolution images of (i) Hoechst-stained nuclei and (ii) Sytox-stained NETs in **(A)** unstimulated and **(B)** PMA stimulated neutrophils at 240 min. Time-point NET quantification using high-content analysis in unstimulated (continuous line) vs PMA-stimulated (dashed line) neutrophils for up to 240 min (scale bars represent 200 μm). **(C)** Quantification of NET structures with the NET Detection algorithm compared with **(D)** nuclear area analysis, **(E)** nuclear intensity analysis, and **(F)** percentage of NET forming events calculated by the Nuclear Decondensation algorithm (*n* = 3).

Alongside the algorithm for NET structure analysis, a further algorithm was developed to acquire information on cells in the early stages of NET formation, based on monitoring changes in nuclear morphology and chromatin decondensation. Monitoring nuclear area and staining intensity provides information on the state of chromatin decondensation and, therefore, can reportedly be used as a quantifiable indicator of the early stages of NET formation (24). The Nuclear Decondensation algorithm provided information on Hoechst-stained neutrophil nuclei and identified as “NET forming” those cells with relatively large areas and low signal intensity compared with unstimulated controls. Neutrophils were stimulated with PMA or were unstimulated (controls) for 3 h (Figures 5A,B). Results shown in Figure 5D indicated the Nuclear Decondensation algorithm in unstimulated neutrophil nuclei calculated an average nuclear area of between 500 and 700 pixels throughout the 4-h incubation period. A similar nuclear area was calculated in PMA-stimulated neutrophils for up to 3 h and increased to above 1,000 pixels being detected after 4 h. Opposing trends were seen for the analysis of nuclear staining intensity. PMA-stimulated neutrophil nuclear intensity was constantly found to be 200 units below the measured intensity in unstimulated neutrophils during the initial 3 h of exposure to PMA and decreased to 500 units at 4 h (Figure 5E). Nuclei which presented simultaneously enlarged areas and a diminished nuclear intensity were subsequently considered by the algorithm as NETotic events. Data presented in Figure 5F indicate the percentage of NET forming events calculated in unstimulated and stimulated neutrophils related to the previously described area and intensity data. The percentage of NET forming events remained at approximately 0% in unstimulated neutrophils, while in PMA-stimulated neutrophils approximately 60% of cells were determined to be in a NETosing state at 4 h exposure to PMA. To further validate and confirm the data obtained with the Nuclear Decondensation algorithm, we analyzed the set of images using the NET Detection algorithm. Interestingly, the trend for NET forming cells calculated with the Nuclear Decondensation algorithm was similar to the trend of NET release calculated using the NET Detection algorithm (Figure 5C): after 4 h exposure to PMA stimulated neutrophils presented higher levels of NET release compared with unstimulated neutrophils. Taken together, these data indicate that monitoring nuclear decondensation provides a valid indicator of NET forming activity and directly relates to NET formation.

### High-Throughput Screening of NETosis Modulators

The HCA method described above was applied for HCS of a pharmaceutically derived compound library to identify modulators of NETosis. Celastrol-treated cells were used as inhibitory controls while Benzethonium Chloride was used to induce cytotoxicity and to discern NETosis from other forms of neutrophil cell death. After 30 min of compound pre-treatment, neutrophils were stimulated with PMA or left unstimulated for 3 h. Following fixation, Hoechst was applied for nuclear staining and images were acquired using the ArrayScan™. HCA was performed using the Nuclear Detection algorithm and image analysis performed using two distinct methods for unstimulated and PMA-stimulated images. To identify whether compounds were able to induce NETosis in unstimulated conditions, rejection and threshold criteria for nuclear size and nuclear staining intensity were defined based on images of unstimulated and untreated neutrophils. The algorithm subsequently identified cells with increased nuclear size and with decreased nuclear intensity. Concomitantly increased size and decreased staining intensity was calculated by the algorithm and used as an indication of NETosis. Conversely, in order to identify whether compounds inhibited PMA-induced stimulation, rejection and threshold criteria were established based on images of PMA stimulated neutrophils with untreated neutrophils used as controls. In this case, the algorithm determined the proportion of cells with increased or decreased levels of both nuclear size and staining intensity compared with control cells. HCS identified 17 compounds potentially inducing NETosis in unstimulated neutrophils, while 13 compounds were potentially identified as inhibiting NETosis and 13 compounds potentially enhanced PMA-induced NETosis (Figure S1 in Supplementary Material). Rapamycin and Lapatinib treatments resulted in inhibition of NETosis at relatively low doses and increased NETosis at higher doses, while Bosutinib treatment resulted in an opposing effect. The compounds exhibiting modulatory activity in unstimulated and PMA-stimulated neutrophils were selected for further screening.

Images associated with the compounds identified by the initial HCS were further analyzed using the HCS Reader Software. The nuclei in each image were analyzed for false-negative or false-positive events and for apoptosis-related phenotypes as previously identified in neutrophils by Fuchs et al. (4). Although apoptotic bodies were visible as black spots, the nuclei still presented similar features to NETosing activity, which was misinterpreted by the algorithm and, therefore, these were manually excluded from the selection process (data not shown).

**Figure 6:**
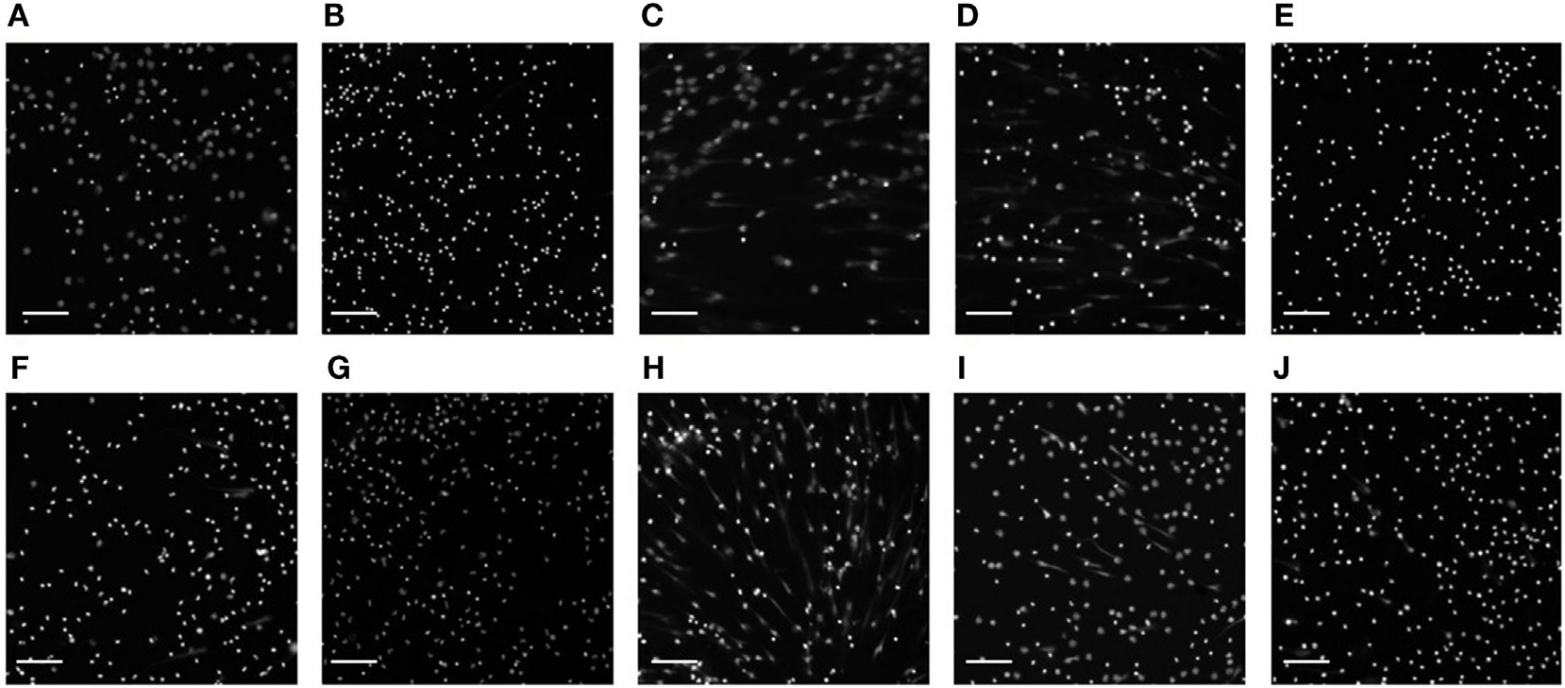
Images of compound-treated neutrophil nuclei. Representative images of 3 h PMA-stimulated neutrophil nuclei **(A)** untreated or **(B)** pre-treated with celastrol and the eight compounds selected [**(C)** Crizotinib; **(D)** Lapatinib; **(E)** Carmustine; **(F)** Erlotinib; **(G)** Bosutinib; **(H)** Ponatinib; **(I)** Rapamycin; **(J)** Nilotinib] using high-content screening and manual screening applied at their highest concentration (10 µM). Nuclei and neutrophil extracellular traps are visible following Hoechst staining. Images are representative of three independent experiments. Scale bars represent 400 µm.

Following extensive literature searching, those compounds that were associated with neutropenia or other forms of neutrophil cell death were also excluded from further study. Subsequently, of the 56 compounds initially screened for modulation of NET formation, 8 were selected for further downstream characterization (listed in Table 1) as NET-inducers, inhibitors or with a biphasic modulatory activity. Images in Figure 6 show PMA stimulated and compound-treated neutrophils. PMA stimulated nuclei (Figure 6A), utilized for the determination of nuclear size and intensity thresholds, presented a characteristic NETosing morphology. In Figure 6B, Celastrol-treated neutrophils, as expected, exhibited nuclei with characteristically unstimulated features and represented controls for inhibitory activity (33).

**Table 1:** Summary of the eight selected neutrophil extracellular trap (NET) modulating compounds, their known molecular targets and induced nuclear phenotype.

Compound	Target of inhibition	Induced nuclear phenotype	Type of modulator
Crizotinib	Anaplastic lymphoma kinase/Proto-oncogene 1 receptor (ROS1) tyrosine kinase (TK) (37)	Decondensed state and visible extracellular DNA (Figure 6C)	NETosis inducer
Lapatinib	Human epidermal growth factor receptor/epithelial growth factor receptor (EGFR) TK (38)	Decondensed state at high dosage (Figure 6D) and condensed state at low dosage (data not shown)	Biphasic (inhibition at low dosage and induction at high dosage)
Carmustine	Glutathione reductase (39)	Condensed state (Figure 6E)	NETosis inhibitor
Erlotinib	Epithelial growth factor receptor (EGFR) TK (40)	Condensed state (Figure 6F)	NETosis inhibitor
Bosutinib	Src family kinases (41)	Condensed state at high dosage (Figure 6G) and decondensed state at low dosage (data not shown)	Biphasic (induction at low dosage and inhibition at high dosage)
Ponatinib	Vascular endothelial growth factor receptor/fibroblast growth factor receptor TK (42)	Decondensed state and visible extracellular DNA (Figure 6H)	NETosis inducer
Rapamycin	Mammalian target of rapamycin/hypoxia-inducible factor 1α (32)	Decondensed state at high dosage (Figure 6I) and condensed state at low dosage (data not shown)	Biphasic (inhibition at low dosage and induction at high dosage)
Nilotinib	Platelet-derived growth factor receptor TK (43)	Condensed state (Figure 6J)	NETosis inhibitor

### Neutrophil Dose–Responses to Selected Compounds

The eight selected compounds were characterized for their neutrophil modulatory activity using more targeted approaches. Compounds were simultaneously tested in neutrophils from three donors at a range of concentrations from 1 nM to 10 µM. PMA treatment was used as a positive control for stimulation, while Celastrol and media alone were used as a negative control and to provide baseline readouts, respectively. Intracellular and extracellular ROS productions were monitored by chemiluminescence assay. Simultaneously, their ability to induce NETosis was evaluated by fluorometric quantification.

Neutrophil ROS production was evaluated for 3 h following compound treatment. PMA stimulation effectively induced ROS production, while data indicated none of the eight compound treatments induced the activation of the ROS cascade (Figure S2 in Supplementary Material). Celastrol treatment resulted in statistically significant lower levels of ROS compared with media control, indicating the compound-driven inhibition may also suppress the normal metabolic activity of unstimulated cells. Similar statistically significant differences were detected with Bosutinib and Ponatib treatments at relatively high doses (respectively, at 10 µM and at 1 and 10 µM), indicating the two compounds exert inhibitory effects similar to that of Celastrol.

**Figure 7:**
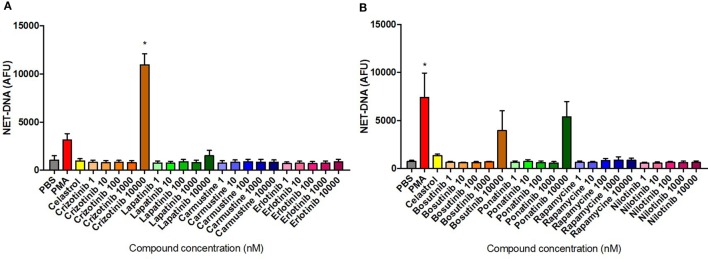
Neutrophil extracellular trap (NET) production quantified in compound-treated neutrophils. Fluorometric NET quantification at 3 h in unstimulated neutrophils untreated or pre-treated with 10 µM Celastrol or the eight selected compounds using concentrations ranging from 1 nM to 10 µM [**(A)** Crizotinib, Lapatinib, Carmustine, Erlotinib; **(B)** Bosutinib, Ponatinib, Rapamycine, Nilotinib], or stimulated with 50 nM PMA. Values are expressed as mean arbitrary fluorescence units (AFU) ± SEM. Statistical significance was calculated using the Kruskal–Wallis’s multiple comparison test (*n* = 3; *p < 0.05 compared with PBS control values).

Dose–response induction of NET formation in unstimulated neutrophils was evaluated by fluorometric measurement of extracellular NET-DNA release following 3 h treatment with the selected compounds. Among the eight compounds tested, only Crizotinib treatment at 10 µM induced a significantly higher amount of NET-DNA release compared with the unstimulated/untreated control (Figure 7A). Furthermore, Bosutinib and Ponatinib treatments at 10 µM resulted in increased NET-DNA release, although the difference compared with the media control was not statistically significant (Figure 7B). The remaining five compounds exhibited similar NET-DNA levels to that of the untreated control indicating their application to neutrophils did not trigger NET formation. Notably, the data for Crizotinib and Ponatinib treatments were consistent with the NET-inducing activity as identified by HCS.

### Compound Dose–Response Modulation of PMA-Induced Neutrophil Activation

The eight selected compounds were characterized for their ability to modulate responses in PMA activated neutrophils from three donors. Neutrophils were treated with the selected compounds at a range of concentrations from 1 nM to 10 µM and incubated for 30 min prior to PMA stimulation for up to 3 h. Treatment with PMA only was utilized as the positive control, while Celastrol treatment was used as a control for inhibitory activity.

**Figure 8:**
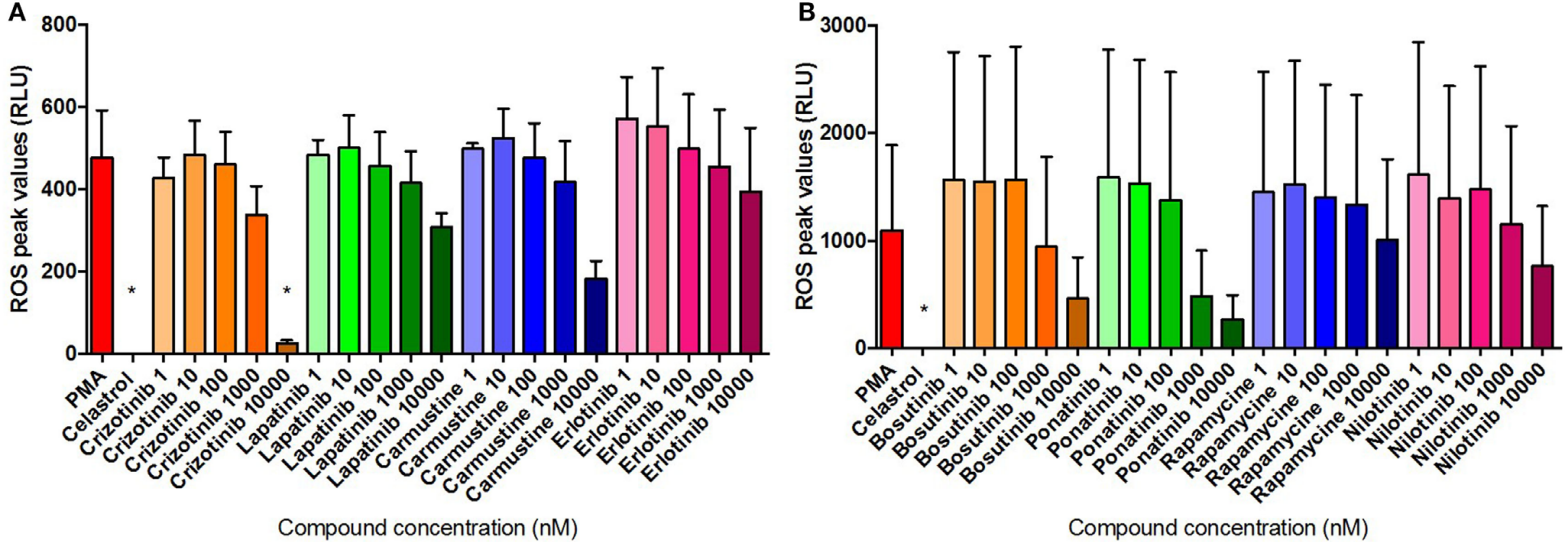
Compound inhibition of PMA-induced ROS. ROS quantification in neutrophils stimulated for 3 h with 50 nM PMA and untreated or pre-treated with 10 µM Celastrol and the eight selected compounds at concentrations of between 1 nM and 10 µM [**(A)** Crizotinib, Lapatinib, Carmustine, Erlotinib; **(B)** Bosutinib, Ponatinib, Rapamycine, Nilotinib]. Values are normalized to the media control and expressed as mean relative light units (RLU) ± SEM. Statistical significance was calculated using the Kruskal–Wallis’s multiple comparison test (*n* = 3; *p < 0.05 compared with PMA control values).

The dose–response modulation of PMA-induced ROS release was monitored for 3 h and the maximum levels of ROS production following treatment were compared with the maximum levels registered in untreated cells throughout the stimulation period. Data presented in Figure 8 were normalized using the media-only control values in order to minimize the physiological differences between donors. Consistently, Celastrol treatment significantly reduced PMA-induced ROS activity. Results presented in Figures 8A,B show an overall dose–response inhibition of ROS production following compound treatment compared with PMA-stimulated neutrophils, although, in general, these results were not statistically significant. Interestingly, Crizotinib treatment at the highest dose (10 µM) was able to significantly reduce the amount of ROS produced in response to PMA stimulation (Figure 8A). Notably, Carmustine, Bosutinib, and Ponatinib treatments, all at the highest dose (10 µM), also reduced levels of PMA-induced ROS (Carmustine in Figure 8A, Bosutinib and Ponatinib in Figure 8B). These differences were, however, not statistically significant.

**Figure 9:**
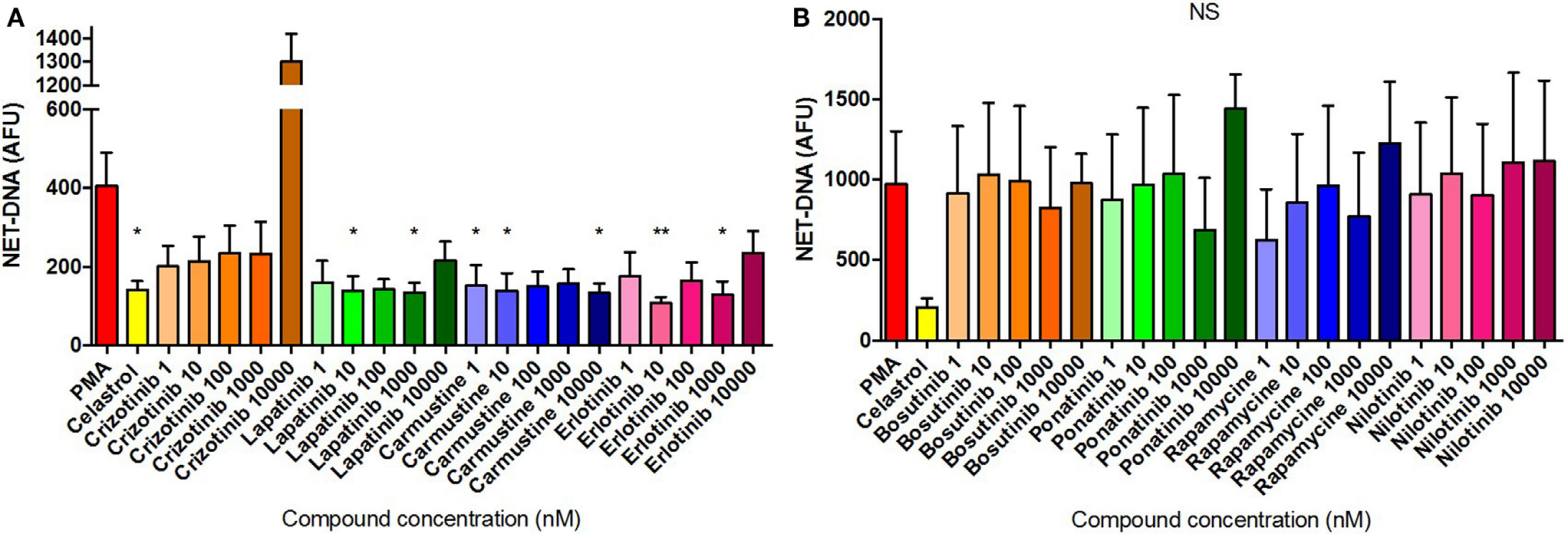
Compound inhibition of PMA-induced neutrophil extracellular traps (NETs). NET quantification in neutrophils stimulated for 3 h with 50 nM PMA and untreated or pre-treated with 10 µM Celastrol and the eight selected compounds at concentrations of between 1 nM and 10 µM [**(A)** Crizotinib, Lapatinib, Carmustine, Erlotinib; **(B)** Bosutinib, Ponatinib, Rapamycine, Nilotinib]. Values were normalized to the media control and expressed as mean arbitrary fluorescence units (AFU) ± SEM. Statistical significance was calculated using the Kruskal–Wallis’s multiple comparison test (*n* = 3; ns = non-significant, *p < 0.05, **p < 0.01 compared with PMA control values).

The dose–response modulation of PMA-induced NETosis by different compounds was evaluated by fluorometric measurement of extracellular NET-DNA release following 3 h incubation. Data presented in Figure 9 were normalized by media control values in order to minimize physiological differences between donors. Inhibition of NET release was identified following treatment with Lapatinib at 10 nM and 1 µM; Carmustine at 1 nM, 10 nM, and 10 µM; and Erlotinib at 10 nM and 1 µM (Figure 9A). Interestingly, Crizotininb treatment at the highest dose (10 µM) resulted in more than threefold higher DNA release, although this was not statistically significant, compared with the untreated control (Figure 9A). Notably, these NET-modulation findings were consistent with their activity as identified by HCS. Statistical significance was identified for the inhibitory effects presented in Figure 9A. Notably, Ponatinib appeared to increase the PMA-induced NET production at higher doses (10 µM), while Rapamycin inhibited NET release at the lowest dose (1 nM) applied (Figure 9B). These differences were, however, not statistically significant.

## Discussion

Several automated and high-throughput techniques have previously been proposed for NET analysis (21, 23, 24, 44), however, many of those approaches exhibit a number of shortcomings including lack of/limited reproducibility and the limitation of single parameter analysis. For the current studies, a protocol was developed and optimized for the analysis of NET formation using HCA. The main advantage of this approach is the potential to analyze a large population of neutrophils contained in the experimental sample, rather than limiting analyses to a selected proportion of the sample population.

A suitable protocol for the preservation of NET samples was developed to enable the use of HCA. Particular attention was paid to optimizing the fixative procedures required for sample preservation, since NETs are fragile structures requiring maintenance of their integrity for accurate analysis (7). The majority of fixation protocols tested were relatively aggressive and caused loss of NET filaments and alterations in the spatial organization of NETs. This property may have contributed to issues identified in previously published NET studies, which largely employed sample preparation on glass slides and used microscopic observation for analysis (45). Subsequently, there is a need for standardization of objective analytical protocols, which are automated and reproducible for NET analysis (21). Once suitable conditions for NET analysis were identified and the fixation protocol was optimized for NET preservation, HCA was performed to quantify the associated features of NET formation and release. The software employed for HCA automated image analysis is designed with a number of built-in algorithms suitable for a wide range of biological applications. The settings of two of these algorithms had characteristics that were deemed appropriate for the features of NET formation. These were assessed for their ability to identify neutrophil nuclei and the quantification of NET-related features. However, results obtained using these algorithms indicated that their application for NETosis exhibited key limitations, mostly due to the inaccurate identification of the characteristic web-like morphology of NETs. Two additional algorithms were, therefore, specifically developed for the analysis of NETosis-related features.

The HCA method and the Nuclear Decondensation algorithm optimized for NET analysis were applied for the screening of a compound library, with the aim of identifying modulators which could increase or reduce NET formation and which, therefore, may have potential in therapeutic applications. The library employed was originally developed for cancer cell treatments and comprised a wide range of pharmaceutically relevant compounds with varied targets for immune cell modulation. Following the initial HCS, further manual screening approaches confirmed the identification of eight modulators of NETosis among the original 56 compounds tested. These eight compounds were sub-categorized based on their effects on NET formation as being inducers, inhibitors, or biphasic modulators. Notably, the eight compounds have been found to modulate multiple intracellular pathways in cancer cells and those relevant to neutrophil or NETosis activity are summarized in Table 1. Several of these compounds have been previously associated with NET forming pathways. Indeed, Carmustine’s inhibitory activity has been shown to target glutathione reductase (39). This enzyme catalyzes the reduction of glutathione (GSH) from its oxidized state (GSSG) and is responsible for maintaining a balanced redox state in neutrophils (46). Interestingly, elevated levels of GSSG have been related to the hyperactivity of neutrophils from periodontitis patients (47) and Yan et al. (48) demonstrated that glutathione reductase activity, despite having an antioxidant function, is essential for the production of murine NETs. Recently, a further study demonstrated that glutathionylation is involved in the production of NETs using murine models (49). Furthermore, Bosutinib is an inhibitor of the Src family kinases which have been previously identified as playing a role in regulating yeast-induced NET production (50, 51) Rapamycin has also been previously shown to regulate NETosis through the mTOR pathway and the induction of the hypoxia-inducible factor-1α (HIF-1α) (32, 52, 53).

Other potentially novel signal transduction pathways regulating NETosis were highlighted by the effects of the other compounds and identified roles for several receptor-associated tyrosine kinases. Interestingly, data presented here indicate that the MAPK pathway strongly associates with NET formation. These data are consistent with the findings of previous studies (44, 54, 55) and could represent an interesting target for the treatment of NET-related diseases, since MAPK-driven phosphorylation controls a wide-range of pro-inflammatory signaling responses. Further investigation is, therefore, required to understand the molecular association between NETosis and the pathways identified by HCS and these have the potential to be explored in more detail using the HCA platform. As the identification of NETosis regulators might have a significant impact for the treatment of high-risk patients, future research should focus on NET-related pathways and identifying targets for therapeutic applications. Indeed, future work could employ HCA for the screening of a larger and more targeted compound library, potentially focusing on the MAPK pathway as well as ones containing neutrophil receptor ligands. Such data would provide a better understanding of the regulation of NET formation and identify further compounds with therapeutic utility.

The effects of the eight compounds selected by HCS were further explored using specific low-throughput analysis of ROS and NET production (summarized in Table S2 in Supplementary Material). Compound treatment was assayed in unstimulated and stimulated neutrophils. Notably, data obtained using three different donors were variable and this likely affected the statistical significance of the findings presented here. Indeed donor variability identified by low-throughput analysis of NETs here and by previous publications (21, 56) may be overcome in future studies by the application of the HCA method in which levels of activation are standardized by baseline and maximum activation status using the algorithmic image analysis. Furthermore, using HCA, a greater number of individual donor samples can be simultaneously analyzed adding to the study power.

It can be postulated that a delicate equilibrium exists between NET formation and removal in order to ensure both the adequate protection against infection and, at the same time, avoiding exposing the host tissues to injurious NET content for a prolonged time period. Indeed, when this equilibrium is lost, an unbalanced immune response results in inflammation and disease progression (14). Anti-inflammatory drugs have previously been tested on neutrophils in order to inhibit NET formation (30, 31), however, interestingly, in this study, modulators with both effects, of inducing and inhibiting NETosis, were identified. The identification of compounds that exhibit a broad range of modulatory activity on NET formation offers the potential to establish a homeostatic balance in patients affected by NET-associated diseases. Notably, Crizotinib, Bosutinib, and Ponatinib, were identified as NET inducers and may, therefore, have application for treating NET-deficiency in patients such as those with CGD (4); indeed and importantly, data indicate that their intracellular targets modulate NET formation downstream of the NADPH-Oxidase components. Furthermore, Bosutinib and Ponatinib together with Celastrol, efficiently decreased baseline and PMA-induced ROS production, suggesting treatment with these compounds may have therapeutic application for the hyper-responsiveness of neutrophils from patients affected by periodontitis (35, 57). Compounds identified as NET inhibitors, including Lapatinib, Carmustine, and Erlotinib, may be employed for the treatment of conditions in which excess or aberrant NET production is related to disease development such as CF, SLE, and RA (8, 12, 58). The pathways described above, however, require further investigation to identify the specific molecular mechanism behind their modulation of NET production.

## Ethics Statement

This study was carried out in accordance with the recommendations of the West Midlands Research Ethics Committee with written informed consent from all subjects. All subjects gave written informed consent in accordance with the Declaration of Helsinki. The protocol was approved by the West Midlands Research Ethics Committee (Ethical approval number: 10/H1208/48).

## Author Contributions

IJC contributed to research design, performed the experiments, was responsible for the analysis and interpretation of data, and wrote the manuscript. MRM, ILCC, GG, RB, and PRC contributed to the conception and design of the study, and assisted with the interpretation of data. GG and RB provided the compound library and assisted with HCA image and data analysis. MRM, ILCC, TD, and PRC critically read and contributed to revisions of the manuscript and approved the final version of the manuscript.

## Conflict of Interest Statement

I.J.C. received research funding from Imagen Therapeutics Ltd.; G.G. and R.B. own shares in Imagen Therapeutics Ltd.; The funding organisation played no role in the study design; in the collection, analysis, and interpretation of data; in the writing of the report; or in the decision to submit the report for publication. The reviewer IN and handling Editor declared their shared affiliation.
