# Supplementary material for: Development and Application of High-Content Biological Screening for Modulators of NET Production
Source: Front Immunol. 2018 Mar 5;9:337. doi: 10.3389/fimmu.2018.00337 (PMC5844942; doi:10.3389/fimmu.2018.00337)
Supplement: Supplementary file 1 [file image_1.PDF]

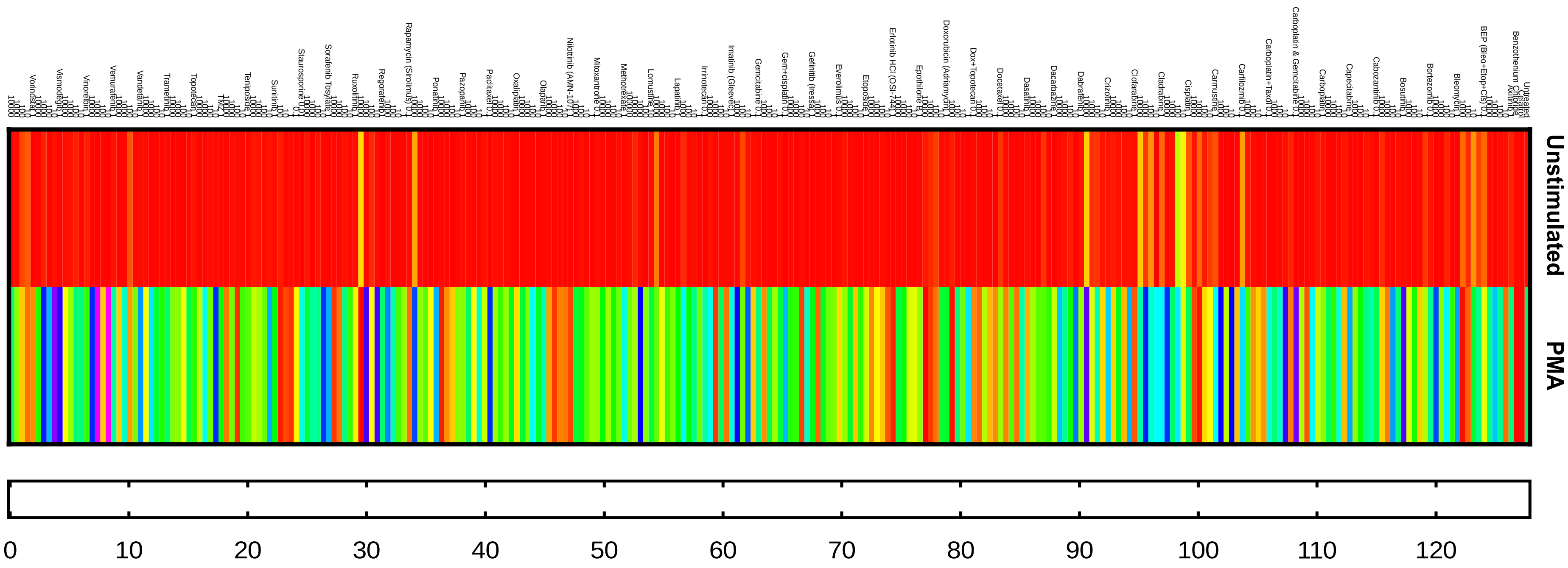

Figure S1: Heatmap representation of the range of activities induced by the compounds on NETosis.

Heatmap representing the NETotic events calculated by HCS in unstimulated and PMA-stimulated neutrophils untreated or pre-treated with members of the 56-compound library. Values are calculated as the mean of two independent experiments and are expressed in a colour-based scale where red represents no NET detection and pink represent the maximal NETotic activity registered in the experiment. The untreated condition is represented as red due to the absence of stimulation and green represents PMA-stimulated neutrophils.
