# Supplementary material for: Development and Application of High-Content Biological Screening for Modulators of NET Production
Source: Front Immunol. 2018 Mar 5;9:337. doi: 10.3389/fimmu.2018.00337 (PMC5844942; doi:10.3389/fimmu.2018.00337)
Supplement: Supplementary file 2 [file image_2.PDF]

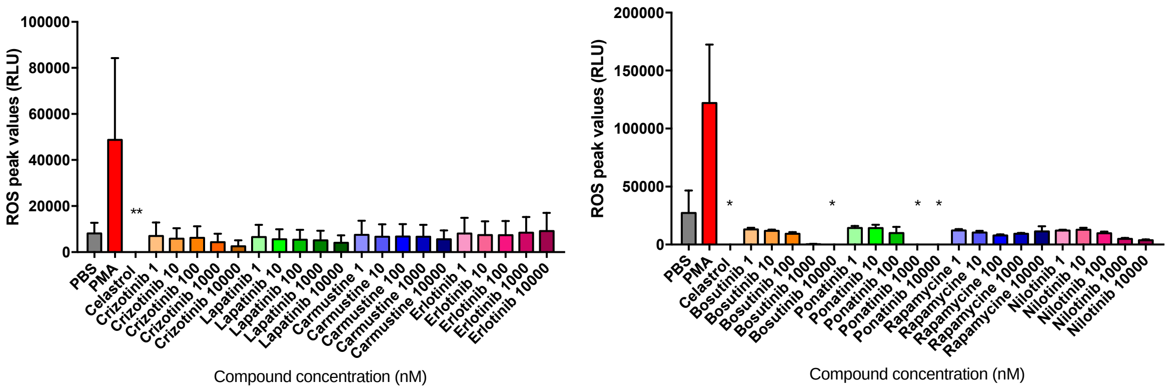

Figure S2: ROS production quantified in compound-treated neutrophils.
ROS production in unstimulated neutrophils which were untreated or pre-treated with 10μM Celastrol or the 8 selected compounds at concentrations ranging from 1nM to 10μM, or stimulated with 50nM PMA for 3 hours. Values are expressed as relative light units (RLU) and mean ± SEM. Statistical significance was calculated using the Kruskal-Wallis's multiple comparison test (n= 3; *p<0.05, **p<0.01 compared with PBS control values).
