# Supplementary material for: Development and Application of High-Content Biological Screening for Modulators of NET Production
Source: Front Immunol. 2018 Mar 5;9:337. doi: 10.3389/fimmu.2018.00337 (PMC5844942; doi:10.3389/fimmu.2018.00337)
Supplement: Supplementary file 3 [file table_1.PDF]

Table S1: Compound library composition
*Combination of equal proportions of specified compounds

Compound name	Dosage	Manufacturer
Benzethonium Chloride	100nM	Selleckchem S4162
Axitinib	1-10000nM	Selleckchem S1005
BEP (Bleomycin, Etoposide, Cisplatin)*	0.1-1000nM	
Bleomycin Sulfate	1-10000nM	Selleckchem S1214
Bortezomib (PS-341)	0.1-1000nM	Selleckchem S1013
Bosutinib (SKI-606)	1-10000nM	Selleckchem S1014
Cabozantinib (XL184, BMS-907351)	0.1-1000nM	Selleckchem S1119
Capecitabine	1-10000nM	Selleckchem S1156
Carboplatin	1-10000nM	Selleckchem S1215
Carboplatin & Gemcitabine*	0.1-1000nM	
Carboplatin & Taxol*	0.1-1000nM	
Carfilzomib (PR-171)	0.1-1000nM	Selleckchem S2853
Carmustine	1-10000nM	Sigma C0400
Cisplatin	1-10000nM	Selleckchem S1166
Cladribine	1-10000nM	Selleckchem S1199
Clofarabine	1-10000nM	Selleckchem S1218
Crizotinib (PF-02341066)	1-10000nM	Selleckchem S1068
Dabrafenib (GSK2118436)	1-10000nM	Selleckchem S2807
Dacarbazine	1-10000nM	Selleckchem S1221
Dasatinib	1-10000nM	Selleckchem S1021
Docetaxel	0.1-1000nM	Selleckchem S1148
Doxorubicin & Topotecan*	0.1-1000nM	
Doxorubicin (Adriamycin)	1-10000nM	Selleckchem S1208
Epothilone B (EPO906, Patupilone)	1-10000nM	Selleckchem S1364
Erlotinib HCl (OSI-744)	1-10000nM	Selleckchem S1023
Etoposide	1-10000nM	Selleckchem S1225
Everolimus (RAD001)	0.1-1000nM	Selleckchem S1120
Gefinitib (ZD-1839, Iressa)	1-10000nM	Selleckchem S1025
Gemcitabine & Cisplatin	0.1-1000nM	

Gemcitabine	0.1-1000nM	Selleckchem S1714
Imatinib (STI571, Gleevec)	1-10000nM	Selleckchem S2475
Irinotecan	0.1-1000nM	Selleckchem S1198
Lapatinib	1-10000nM	Selleckchem S2111
Lomustine	10-100000nM	Selleckchem S1840
Methotrexate	1-10000nM	Selleckchem S1210
Mitoxantrone	0.1-1000nM	Selleckchem S1889
Nilotinib (AMN-107)	1-10000nM	Selleckchem S1033
Olaparib (AZD2281, KU0059436)	1-10000nM	Selleckchem S1060
Oxaliplatin	1-10000nM	Selleckchem S1224
Paclitaxel (Taxol)	0.1-1000nM	Selleckchem S1150
Pazopanib	1-10000nM	Selleckchem S3012
Ponatinib (AP24534)	1-10000nM	Selleckchem S1490
Rapamycin (Sirolimus)	0.1-1000nM	Selleckchem S1039
Regorafenib (BAY 73-4506)	1-10000nM	Selleckchem S1178
Ruxolitinib (INCB018424)	1-10000nM	Selleckchem S1378
Sorafenib Tosylate	1-10000nM	Selleckchem S1040
Staurosporine	0.01-100nM	Selleckchem S1421
Sunitinib	1-10000nM	Selleckchem S7781
Teniposide	1-10000nM	Selleckchem S1787
Temozolomide (TMZ)	1-10000nM	Selleckchem S1237
Topotecan HCl	1-10000nM	Selleckchem S1231
Trametinib (GSK1120212)	1-10000nM	Selleckchem S2673
Vandetanib (ZD6474)	1-10000nM	Selleckchem S1046
Vemurafenib (PLX4032, RG7204)	1-10000nM	Selleckchem S1267
Vinorelbine Tartrate	1-10000nM	Selleckchem S4269
Vismodegib (GDC-0449)	1-10000nM	Selleckchem S1082
Vorinostat (SAHA, MK0683)	1-10000nM	Selleckchem S1047
