# Supplementary material for: Development and Application of High-Content Biological Screening for Modulators of NET Production
Source: Front Immunol. 2018 Mar 5;9:337. doi: 10.3389/fimmu.2018.00337 (PMC5844942; doi:10.3389/fimmu.2018.00337)
Supplement: Supplementary file 4 [file table_2.PDF]

Table S2: Summary of individual compound effects on ROS and NET activities in unstimulated and PMA-stimulated neutrophils.

(none=no effect detected; ns=non-significant)

**Compound**	**ROS**	**NETs**
	**Effect on unstimulated induction (+)/ inhibition (–)**	**Effect on unstimulated induction (+)/ inhibition (–)**
Crizotinib	none	+
Lapatinib	none	none
Carmustine	none	none
Erlotinib	none	none
Bosutinib	–	+ ns
Ponatinib	–	+ ns
Rapamycin	none	none
Nilotinib	none	none
	**Effect on PMA-stimulated induction (+)/ inhibition (–)**	**Effect on PMA- stimulated induction (+)/ inhibition (–)**
Crizotinib	–	+ ns
Lapatinib	none	–
Carmustine	none	–
Erlotinib	none	–
Bosutinib	– ns	none
Ponatinib	– ns	+ ns
Rapamycin	none	– ns
Nilotinib	none	none
